# Synaptic dysfunction in complex psychiatric disorders: from genetics to mechanisms

**DOI:** 10.1186/s13073-018-0518-5

**Published:** 2018-01-31

**Authors:** Xinyuan Wang, Kimberly M. Christian, Hongjun Song, Guo-li Ming

**Affiliations:** 1 Department of Neuroscience and Mahoney Institute for Neurosciences, Perelman School of Medicine, University of Pennsylvania, Philadelphia, PA 19104 USA; 2 School of Basic Medical Sciences, Fudan University, Shanghai, 200040 China; 3 Department of Cell and Developmental Biology, Perelman School of Medicine, University of Pennsylvania, Philadelphia, PA 19104 USA; 4 Institute for Regenerative Medicine, Perelman School of Medicine, University of Pennsylvania, Philadelphia, PA 19104 USA; 5 The Epigenetics Institute, Perelman School of Medicine, University of Pennsylvania, Philadelphia, PA 19104 USA; 6 Department of Psychiatry, Perelman School of Medicine, University of Pennsylvania, Philadelphia, PA 19104 USA

## Abstract

Breakthroughs on many fronts have provided strong evidence to support synaptic dysfunction as a causal factor for neuropsychiatric diseases. Genetic studies have identified variants implicated in novel biological and synaptic pathways, and animal and patient-derived induced pluripotent stem cell-based models have allowed mechanistic investigations of synaptic dysfunction in pathological processes.

## Synaptic function and dysfunction in the brain

Synapses are structural elements that allow electrical or chemical signals to flow from one neuron (presynaptic cell) to the next (postsynaptic cell). Synapses can undergo dynamic modifications in the form of synaptic plasticity, which supports essential brain functions such as learning and memory. Dysregulated synaptic development, properties, and plasticity have been hypothesized to underlie altered neuronal function in complex neuropsychiatric disorders, such as schizophrenia (SCZ) and autism spectrum disorder (ASD). For example, adhesion molecules, such as neurexin (NRXN) at the presynaptic site and its ligand, neuroligin (NLGN), at the postsynaptic site, are central organizing proteins for synapse formation and maintenance. Mutations of *NRXN*, *NLGN*, and *SHANK*, which encodes the stabilizer scaffolding protein SHANK at the postsynaptic site, are risk factors for both ASD and SCZ. Immune system components, such as microglia and complement factor C4, also regulate synapse numbers, and mutations in these pathways are linked to both ASD and SCZ. Here, we focus on recent genetic and mechanistic studies that provide novel insights into synaptic dysfunction in complex psychiatric disorders.

## Genetics of synaptic dysfunction in psychiatric disorders

Despite different endophenotypes and ages of onset, SCZ, ASD, and several other complex psychiatric disorders have a developmental origin and strong genetic contributions. Large consortium efforts have made substantial progress in identifying genetic risk factors and in describing the genetic architecture for these disorders, which suggest a convergence in molecular pathophysiology through synaptic dysregulation. A large genome-wide association study (GWAS) identified 128 independent single-nucleotide polymorphisms (SNPs) spanning 108 distinct genetic loci that are associated with SCZ [1]. Results from this study provided strong evidence to support a polygenic contribution to SCZ and identified multiple risk genes that are directly involved in synaptic transmission and plasticity. Another striking finding was the strong association of the major histocompatibility complex (MHC) region with SCZ. The MHC region encodes proteins that are part of the immune system but its relation to SCZ was unclear. The answer came from the study of the complement component 4 (C4) gene within the MHC region that controls the expression of various C4 alleles. C4A is expressed in proportion to the allelic risk association with SCZ, and C4 regulates synapse elimination, or ‘pruning’, during postnatal neurodevelopment in an animal model [2]. Synaptic pruning fine tunes the number of synapses during development and early adulthood. In humans, the number of synapses in the prefrontal cortex peaks at 15 months of age, and then is gradually reduced via synaptic pruning through late adolescence or adulthood [3]. Children with ASD are thought to have excess synapses due to deficits in pruning, whereas patients with SCZ have fewer synapses overall. Intriguingly, the window for synaptic pruning roughly begins with the age of onset for ASD and ends at the age of onset for SCZ, which suggests that dysregulation of synaptic pruning may be an essential part of the pathophysiology in these disorders.

Whole-exome sequencing and targeted sequencing of candidate genes, in deeply phenotyped cohorts, isolated populations, or family studies, have also revealed rare variants associated with psychiatric disorders. For example, a deleterious variant of *CX3CR1*, which encodes a G protein-coupled receptor that binds the chemokine CX3CL1, was found to be associated with increased risk for both SCZ and ASD [4]. As *CX3CR1* is expressed in microglia only, this provides additional support for the role of immune components in psychiatric disorders.

Genetic convergence has also begun to emerge from analyses of multiple psychiatric disorders. In addition to the genes mentioned above, mutations of large genetic loci, which include 2p16.3/*NRXN1*, 15q13.3, and 22q11.21, are also associated with both SCZ and ASD. Genes in these loci have been implicated in neural developmental processes as well as in synapse formation and plasticity. Together, genomic profiling studies have provided novel insights into psychiatric disorders, such as molecular pathways that directly or indirectly affect synaptic function and plasticity. As SCZ and ASD are complex disorders involving multiple brain regions, a central question remains as to how genetic mutations affect specific neural circuits and contribute to divergent clinical phenotypes.

## How do genetic lesions impact synaptic function and lead to manifestation of psychiatric disorders?

Identification of meaningful phenotypes and the use of appropriate functional assays are important challenges in understanding complex psychiatric diseases. In the past decade, with the development of human induced pluripotent stem cell (iPSC) technology and advanced 2D or 3D differentiation protocols, psychiatric disorders now can be modeled in a dish with disease-relevant cell populations. The most common 2D models rely on cortical or hippocampal neurons differentiated from patient-specific iPSCs. Using distinct differentiation protocols to generate cortical neurons from idiopathic SCZ patients [5] or from patients with a defined mutation at the *DISC1* locus [6], results from two independent studies showed synaptic defects in patient iPSC-derived glutamatergic neurons. Moreover, in both studies large-scale gene expression changes were identified in multiple pathways, including the phosphodiesterase (PDE) pathway, which is known to modulate synaptic function. Multiple PDE family enzymes have been intensively studied for therapeutic development to treat schizophrenia. In an ASD iPSC 2D culture study, patient-derived neurons displayed abnormal neurogenesis and reduced synapse formation, leading to defects in synaptic function and neuronal network activity [7]. Interestingly, IGF-1 treatment rescued the network activity.

Patient iPSC-based 3D human brain organoids have provided additional insight into the biological basis of neuropsychiatric diseases. Consistent with macrocephaly reported in clinical MRI studies of ASD patients, brain organoids derived from idiopathic ASD patient iPSCs exhibited a transient increase in size and faster neural progenitor proliferation when compared with controls [8]. GABAergic neuron production was also increased in a FOXG1-dependent fashion. Another study focused on two well-known SCZ risk genes and demonstrated that specific interruption of the DISC1–Ndel1/Nde1 interaction leads to cell-cycle progression defects in radial glial cells, both in embryonic mouse cortex and human forebrain organoids [9]. The same phenotype is also observed in patient iPSC-derived organoids with a *DISC1* mutation, which disrupts DISC1–Ndel1/Nde1 interaction. These studies suggest that early developmental events, such as neuronal proliferation and differentiation, which precede synaptic development, might also contribute to ASD and SCZ. Considering that each neuron forms many synapses, these early neurogenic events may have a larger net impact on neuronal circuitry.

Several recent studies have shed light on the role of microglia in regulating brain function by synaptic pruning. In mice, loss of PGRN (progranulin), a key regulator of inflammation, leads to increased complement activation and excessive microglia-mediated pruning of inhibitory synapses in the ventral thalamus, which in turn resulted in hyperexcitability in thalamocortical circuits and abnormal grooming behaviors [10]. Autophagy appears to be essential for microglia-mediated synaptic pruning. Deletion of *Atg7* (an autophagy essential gene) specifically in microglia abolishes its ability to prune synapses, resulting in increased synapse numbers, altered brain region connectivity, and ASD-like repetitive behaviors and social behavior defects [11]. Together with genetic findings that implicate immune components in the risk for psychiatric disorders, these studies provide a mechanistic hypothesis for how dysregulated microglia activation leads to synaptic dysfunction.

## Conclusions and future directions

Human genetic studies of psychiatric disorders are picking up pace with increasing cohort sizes and the use of whole-genome sequencing. Large-scale GWA studies of idiopathic patients have shed light on common, low penetrance variants, and mechanistic studies of rare, high penetrance risk genes have deepened our knowledge of disease-relevant biological processes. Importantly, these two types of studies are mutually informative and have revealed convergence at a broad functional level between common and rare genetic variations [5, 6]. Results from these studies highlight the importance and complexity of synaptic deficits in neuropsychiatric disorders. Recent evidence has provided clear support for several mechanistic hypotheses, including: a direct impact of genetic risk factor interactions on neurodevelopment, as in the case of *DISC1–Ndel1/Nde1;* neuronal circuitry and behavioral deficits arising from impaired synaptic pruning; and the involvement of immune system components in the primary pathogenesis underlying psychiatric disorders. Human-specific models at the circuit or system level using 3D brain organoids will need to be developed to further test these hypotheses. Another important question is the role of postzygotic mosaic mutations in neuropsychiatric diseases, which requires analysis of surgical or post-mortem samples of brain tissue. Although access to this tissue is limited, advances in single-cell whole genome sequencing will maximize the information we can acquire from each sample.

Similar to heterogeneous clinical presentations, the etiologies of psychiatric disorders are highly complex. Enriched diagnostic information and patient stratification is essential to develop precise and efficient therapies. Integrating the findings from human genetic analyses, transgenic animal models, and patient-derived stem cell models will accelerate our progress toward better therapeutic targets and strategies, based on an appreciation of the causal biological processes.

## Abbreviations

ASD: Autism spectrum disorder
GWAS: Genome-wide association study
iPSC: Induced pluripotent stem cell
MHC: Major histocompatibility complex
PDE: Phosphodiesterase
SCZ: Schizophrenia
SNP: Single-nucleotide polymorphism
